# M3VR—A multi-stage, multi-resolution, and multi-volumes-of-interest volume registration method applied to 3D endovaginal ultrasound

**DOI:** 10.1371/journal.pone.0224583

**Published:** 2019-11-21

**Authors:** Qi Xing, Parag Chitnis, Siddhartha Sikdar, Jonia Alshiek, S. Abbas Shobeiri, Qi Wei

**Affiliations:** 1 Department of Computer Science, George Mason University, Fairfax, Virginia, United States of America; 2 The School of Information Science and Technology, Southwest Jiaotong University, Sichuan, China; 3 Department of Bioengineering, George Mason University, Fairfax, Virginia, United States of America; 4 Department of Obstetrics & Gynecology, INOVA Health System, Falls Church, Virginia, United States of America; St. Vincent Medical Center, UNITED STATES

## Abstract

Heterogeneity of echo-texture and lack of sharply delineated tissue boundaries in diagnostic ultrasound images make three-dimensional (3D) registration challenging, especially when the volumes to be registered are considerably different due to local changes. We implemented a novel computational method that optimally registers volumetric ultrasound image data containing significant and local anatomical differences. It is A Multi-stage, Multi-resolution, and Multi-volumes-of-interest Volume Registration Method. A single region registration is optimized first for a close initial alignment to avoid convergence to a locally optimal solution. Multiple sub-volumes of interest can then be selected as target alignment regions to achieve confident consistency across the volume. Finally, a multi-resolution rigid registration is performed on these sub-volumes associated with different weights in the cost function. We applied the method on 3D endovaginal ultrasound image data acquired from patients during biopsy procedure of the pelvic floor muscle. Systematic assessment of our proposed method through cross validation demonstrated its accuracy and robustness. The algorithm can also be applied on medical imaging data of other modalities for which the traditional rigid registration methods would fail.

## Introduction

Medical image registration is the process of aligning two or more radiological images in two or three dimensions [1]. It has been widely applied for radiotherapy treatment planning [2], image segmentation [3], image guided surgery [4], and longitudinal monitoring [5] among many other applications. Automated and semi-automated registration methods seek to determine a transformation between two or more image datasets by solving an optimization problem that maximizes the similarity between the transformed datasets. These datasets may be possibly of different imaging modalities, from different subjects, and/or acquired at different times. Registration through optimization has been an active area of research [1, 6–12]. Three-dimensional (3D) volume registration is more challenging than 2D registration due to the increased number of degrees of freedom needed for transformation and the difficulty in quantifying volumetric discrepancy.

A common application of 3D image registration is the longitudinal monitoring before and after an interventional procedure. Ultrasound imaging is attractive for longitudinal monitoring because it is inexpensive and noninvasive. 3D volumetric registration of ultrasound images is considered more challenging than other medical image modalities such as Computed Tomography and Magnetic Resonance Imaging (MRI) [13] because of the low signal-to-noise ratio, heterogeneity of echo textures, and low ultrasound image contrast due to artifacts and attenuation [14]. In particular, ultrasound imaging of soft tissue can be subject to considerable signal to noise ratio variation [14], frequency-dependent attenuation of the backscattered signals [15], and tissue deformation. In addition, interventional procedures, such as needle biopsy, often induce tissue deformation and hematoma [16, 17]. As a result, datasets that were acquired from the same patient before and after an intervention can be considerably different, making registration difficult.

3D volume registration methods can be generally classified into two types: rigid registration using linear transformation and non-rigid registration using nonlinear transformation to compensate for deformation of structures [18, 19]. For example, a rigid transformation that includes translation and rotation can be used to align two volumetric data. When significant differences exist due to natural variations among subjects or changes in physical structures over time such as tumor development or other factors, non-rigid image registration should be applied to allow localized deformation and to optimally align different datasets [20]. For our problem, we chose to solve for a rigid registration because of the overall rigidity of the volume. Non-rigid transformation may introduce too many degrees of freedom. This may potentially overfit the data to heterogeneous ultrasound echo textures and result in undesired soft tissue deformation.

Existing 3D ultrasound registration methods are either feature-based [21–24], intensity-based [25–29], or combination of the two [30, 31]. Feature-based methods rely on identifiable features that are extracted from ultrasound image. These features can be anatomical landmarks, structure boundaries, surfaces, or statistical shape features. Many 3D feature-based methods have been developed. A few representative ones include a surface-based, nonlinear registration method to align ultrasound images to histological data in 3D [21], a temporal and spatial registration approach to align pre-and post-stress ultrasound images [22], a novel method based on spatial Gabor texture similarity represented by statistical kernel distributions [23] and registration between an ultrasound volume and a statistical shape model using local phase bone features from ultrasound images [24]. 3D scale- and rotation-invariant key points have been used to identify 3D features in fixed and moving volumes [32, 33]. However, definition and matching of these key points can be difficult.

Compared to intensity-based methods, feature-based approaches are recognized to be more robust to intensity variation and less susceptible to local minima [30], however require additional steps on feature extraction. Intensity-based methods [25–29, 31, 34, 35] use raw pixel intensity values to assess similarity between two datasets and do not need additional features. One recently proposed intensity-based registration approach involves block-matching and an outlier rejection method to determine an affine transformation between ultrasound volumes [25]. Mutual information-based methods are commonly used in registering ultrasound volumes [28, 29]. An intensity-based deformable registration method was employed for 3D ultrasound registration through a variational framework [34]. Intensity-based approaches might be computationally extensive and converge to local minima.

Existing registration approaches [9, 25, 32–34, 36–38] are not suitable to produce precise alignment under the challenging conditions mentioned above. As such, there is a need to develop new automated volumetric registration methods. Globally rigid registration will likely fail to find the optimal alignment because the anatomical changes obviously violate the rigidity assumption. Contrarily, non-rigid registration may strive to accommodate those changes but may result in undesirable deformation in other regions due to the many degrees of freedom associated with these methods. In this study, we developed and validated a 3D ultrasound registration method that overcomes these challenges.

Our proposed method is called M3VR, for Multi-stage, Multi-resolution, Multi-volume of interest Volumetric Registration. It is a two-stage, weighted multiple volume-of-interest (VOI) registration method. M3VR can be applied on volumetric data of any medical imaging modality that contain rigid non-deformable structures such as bone as well as deformable tissue such as muscle. In the first stage, a rigid registration is applied on a single VOI containing rigid structures to find initial alignment. In the second stage, multiple VOIs that are distributed across the whole volume and do not include the anatomically different regions are selected to add influence of soft tissue structures. Then a weighted multiple VOI registration is performed to calculate the optimal alignment of all these VOIs in order to refine the alignment obtained from the first stage.

**Fig 1:**
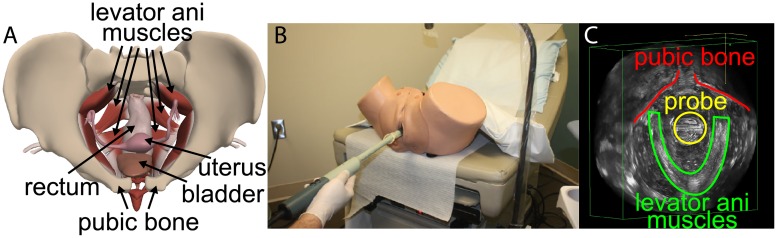
The anatomy of the women pelvic floor. (A) Axial view. Pelvic floor muscles are the layer of muscles that support pelvic floor organs, assist in urinary and fecal continence and stabilize connecting joints. (B) 3D Endovaginal Ultrasound Imaging with 360° rotational transducer using a BK Ultrasound System (Flex Focus 500) [9]. (C) Pelvic floor muscle appears as a V-shaped structure in 3DEVUS (green boundary). Pubic bone and probe can also be viewed interactively during acquisition and offline.

We evaluated our M3VR in a 3D Endovaginal Ultrasonography (3DEVUS) application for assessing and monitoring pelvic floor disorders, which affect nearly 24% of US women [39, 40]. Pelvic organ prolapse (POP) is a common type of pelvic floor disorder in which the pelvic organs (bladder, uterus and rectum) descend downward due to the loss of mechanical integrity of the pelvic floor (Fig 1A), particularly that of the levator ani muscle and the associated connective tissues [41]. POP is associated with significant dysfunction and detrimental impact on the quality of life of these patients. Injury to the levator ani muscle during vaginal birth is a significant risk factor for developing POP [42]. The effects of these injuries can be additive with subsequent births and can result in levator ani muscle atrophy and increased risk of POP [42, 43]. Accurate and timely detection of injury to the levator ani muscle is critical to the effective treatment of POP. Modern imaging techniques such as MRI and ultrasonography are currently used clinically in the non-invasive examination of levator muscle injury [39, 42, 44–48].

Compared to MRI, ultrasonography has the advantage of being fast, cost effective, and easily available. 3DEVUS provides a comprehensive overview of the pelvic floor anatomy by performing a 360° field-of-view scanning (Fig 1B and 1C). Quantitative analysis of the progression or repair of muscle injury requires longitudinal monitoring and volumetric registration of the acquired 3DEVUS images [49].

In this paper, we first describe the pelvic floor data used in this study and the M3VR method in Section 3. Validation metrics and procedure are also discussed. Section 4 presents our results on the application of M3VR on 3D endovaginal ultrasound data, parameter determination, as well as validation of the results. In Section 5, we discuss limitations of the proposed method and future directions of this work.

## Methods

### Ultrasound imaging of the pelvic floor

**Fig 2:**
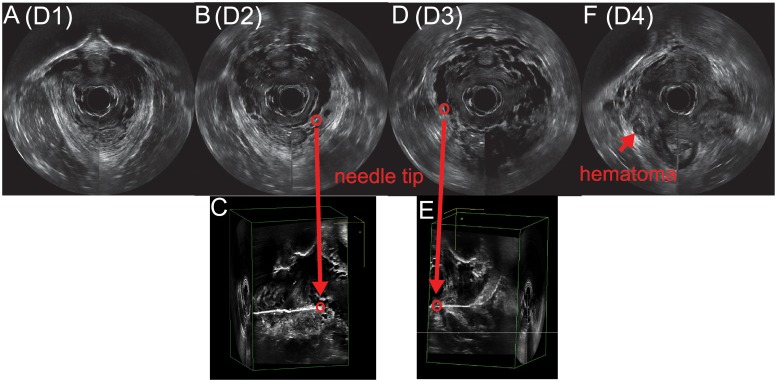
Four phrased 3DEVUS data collected from the same subject during biopsy examination. (A) Pre-biopsy 3DEVUS volume. (B) 3DEVUS volume with biopsy needle on the left. (C) Needle inserted on the left is more visible from a different view. (D) 3DEVUS volume with the biopsy needle on the right. (E) Needle inserted on the right viewed from the sagittal view. (F) Post-biopsy 3DEVUS volume. The development of hematoma can clearly be seen as indicated by the arrow, which showed up as a dark region at the biopsy site.

**Fig 3:**
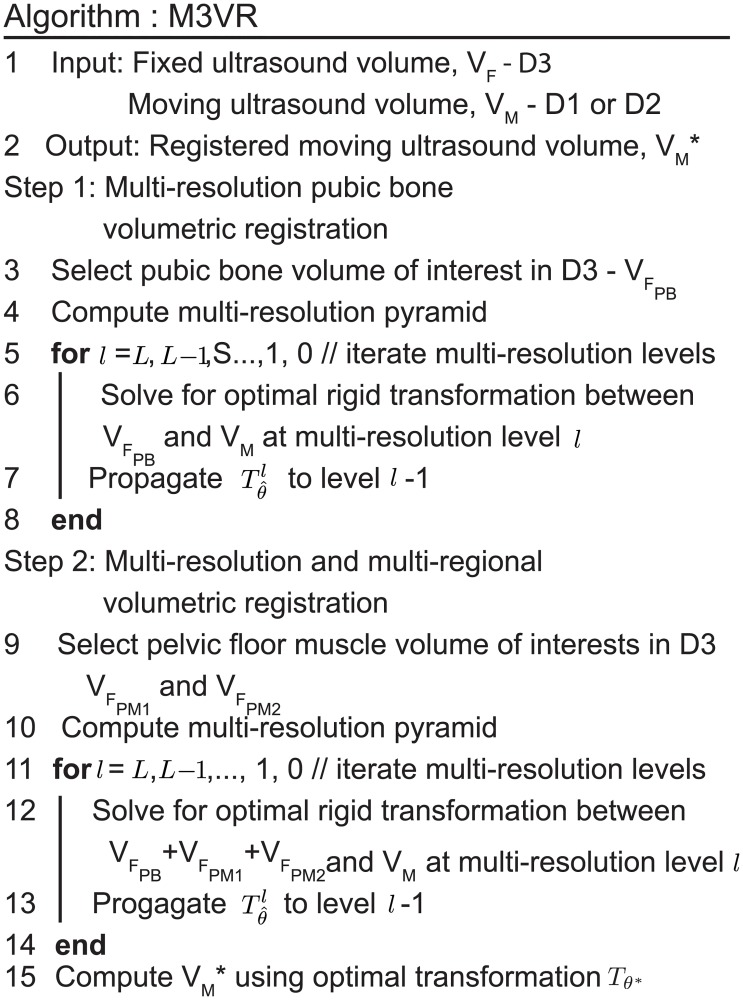
Algorithm of M3VR. Two-stage multi-resolution volume registration. Stage 1: pubic bone sub-volume registration. Stage 2: spatially weighted multiple sub-volumes registration on pubic bone and pelvic floor muscles.

**Fig 4:**
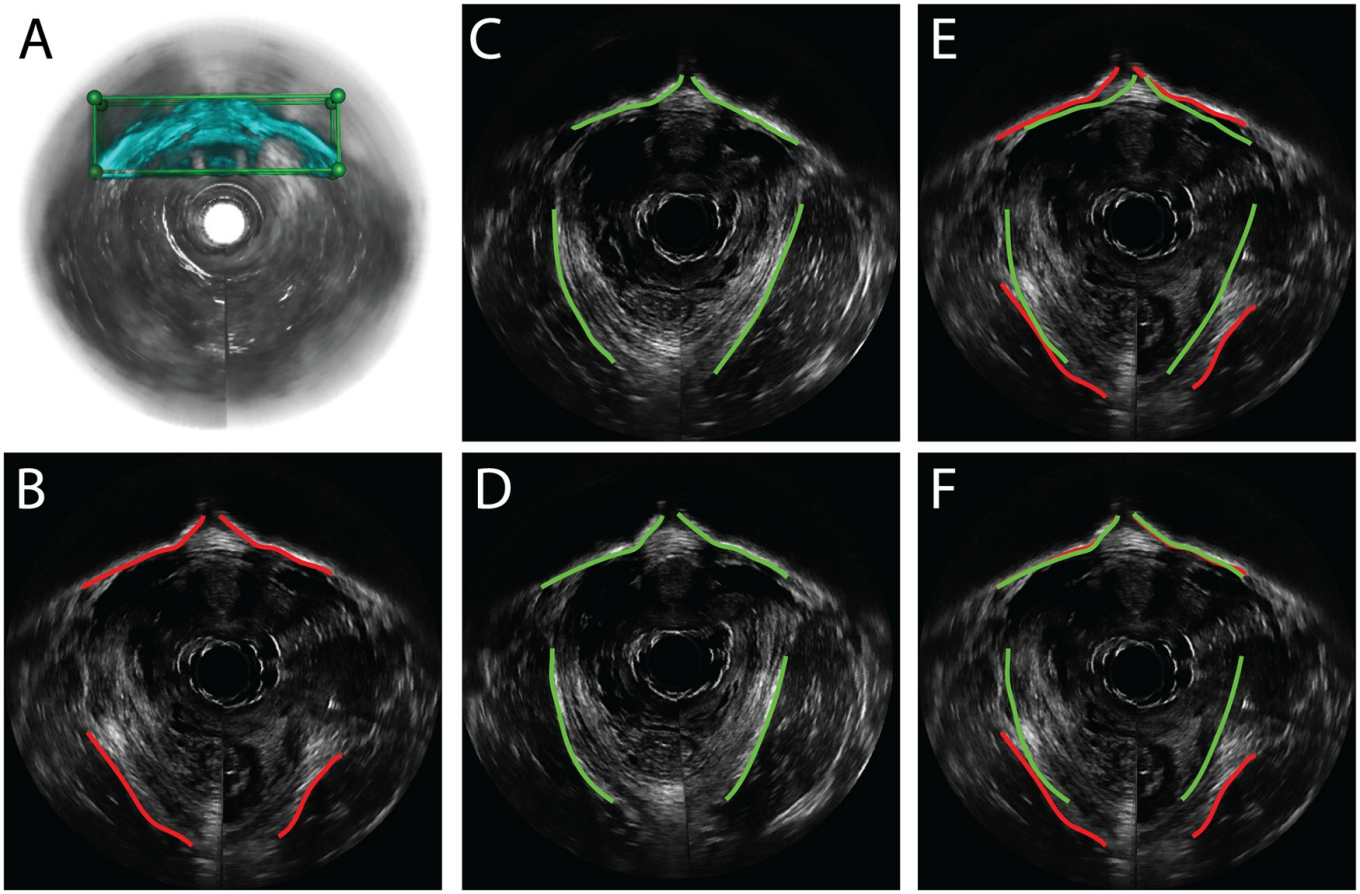
Intensity-based volumetric registration on pubic bone VOI. (A) A cubic VOI enclosing the public bone was defined in the fixed image volume (green grid). The pubic bone was rendered in cyan simply to highlight the bone dimensions. (B) One slice image from fixed image volume with pubic bone and muscle boundaries. (C) Corresponding image slice before registration in the moving image volume. (D) Corresponding image slice after registration in the transformed moving image volume. (E) Pubic bone and muscle boundaries comparison before registration, the initial boundaries are not matched between fixed and moving images. (F) Pubic bone and muscle boundaries comparison after registration. The pubic bone boundaries are better aligned demonstrating the effectiveness of first stage registration.

As part of a previous study, we collected 3DEVUS data from subjects who had different degrees of levator ani muscle injury and underwent biopsy [50]. 3DEVUS was performed on each patient once before, twice during, and once after the biopsy (Fig 2). The four datasets (volume size: 94mm × 94mm × 65mm; spatial resolution: 0.118mm/pixel) per patient include: (1) a pre-biopsy ultrasound volume (D1 in Fig 2A) with muscle intact for pre-operative examination, (2) an ultrasound volume with biopsy needle positioned on the left side of the pelvis (D2 in Fig 2B), (3) an ultrasound volume with biopsy needle positioned on the right side (D3 in Fig 2D), and (4) a post-biopsy ultrasound volume (D4 in Fig 2F). D2 and D3 were obtained during biopsy to guide placement of the J-hook needle in the pelvic floor muscle region with suspected muscle defect. This technique was similar to that of a previous study by Busacchi et al. [51].

The proposed study involved secondary analysis of 3DEVUS data collected previously from patients who had different degrees of levator ani muscle injury and underwent biopsy. The previous study was approved by the Institutional Review Board at the University of Oklahoma in 2012 enroll 85 patients who were scheduled for surgery. Informed consent were obtained from all participants who met the eligibility criteria. Ultrasound volumes obtained from research and clinic patients were kept on secure servers. We also maintained a database of this population on a secure server. A data share agreement was made among the University of Oklahoma, Inova, and George Mason University for secondary analysis of the data. The data was anonymized by Dr. Shobeiri before being presented to the rest of the team so that the US volumes were analyzed blindly to other patient information.

The long term goal is to apply quantitative ultrasound analysis to assess pelvic floor muscle injury, which correlates muscle characteristics obtained from statistical ultrasound texture analysis at the biopsy location to those obtained from histologic analysis from the needled biopsy sample. The biopsied tissue was not visible in the ultrasound volume obtained during biopsy because of the needle. However, it was completely visible and intact in the pre-biopsy data. In order to establish an accurate correlation, the needle location needs to be determined in the pre-biopsy data such that quantitative ultrasound analysis can be performed on the exact biopsied tissue. A precise spatial registration method that optimally aligns the ultrasound volumes with needle inside (D2 and D3) to the volume without needle (D1) is required to objectively estimate biopsied tissue location. Although these volumetric data were acquired from the same subject, optimal alignment is not a trivial problem. D1, D2 and D3 were taken at different time during the procedure with ultrasound probe repositioned for each dataset. There was unavoidable displacement of the probe by the clinician, which caused tissue deformation near the probe and variable transformation of the imaged volume. These volumetric data were also subject to significant tissue change due to insertion of the needle and development of post-biopsy hematoma.

### M3VR registration framework

The M3VR framework is shown in Fig 3. D3 is defined as the fixed ultrasound volume *V*_*F*_. D1 and D2 are defined as the moving ultrasound volumes *V*_*M*_, which are to be aligned with the fixed volume D3. In the first stage, an intensity-based multi-resolution rigid registration method was performed on the pubic bone sub-volume to provide an initial alignment of the moving ultrasound volumes (D1 and D2) to the fixed ultrasound volume (D3).

As shown in Fig 1C, the pubic bone is located only in the upper half of the volumetric data. Registering the whole volume based on matching a much smaller sub-volume may be subject to bias because of the heterogeneous characteristics of ultrasound imaging and offset of the sub-volume from the center. The resultant error would have been the most severe at places far from the sub-volume. A related issue is the bias of fit problem that has been examined previously in the computer vision community [52]. Nevertheless, such locally targeted registration can effectively bring the two datasets closer to each other, which facilitates subsequent and more sophisticated registration.

In the second stage, spatially weighted, intensity-based, multiple VOIs, and multiple resolution registration was performed to optimize the alignment between transformed D1 and D2. The sub-volume ultrasound data at the biopsy site can be obtained from the transformed needle location for further analysis. We performed extensive validation to ensure the robustness and accuracy of the proposed method. The whole registration pipeline was implemented in C++ and ITK [53].

The M3VR method optimizes alignment by combining multiple volumes of interest that accommodate different anatomical structures. The main idea is to select multiple sub-volumes that do not contain changed anatomical structures. If these sub-volumes are dispersed over the volume, the bias of fitting is unlikely to occur. The pixel intensity values of different anatomical structures may have different ranges. This needs to be considered in the overall similarity measurement. For instance, bone appears brighter than muscle in ultrasound images and thus has higher intensity values. To adjust the contributions of different structures in the combined cost function to enforce either fairness or preference of alignment, weights are applied on the selected volumes of interest to reflect their significance. We will now describe the methodology in detail.

### Stage 1: Multi-resolution pubic bone volumetric registration

In the first stage, we apply the intensity-based volumetric registration method to find the matching homologous regions across the fixed and moving ultrasound volumes demonstrated in Fig 4. Considering the pubic bone region as the most rigid and consistently textured structure in the imaged region, we first calculate the initial transformation based on a volume of interest containing the pubic bone (Fig 4A). The VOI is first defined by a 3D bounding box of the pubic bone in D3 and is considered fixed (Fig 4A). A 3D rigid transformation *T*_*θ*_ was computed by optimizing the registration cost function Eq (1) so that another dataset (D1 or D2) is optimally aligned with D3.

$$\hat{\theta }\;=\;\underset{\theta }{\text{argmin}}\;\phi \left(V_{F},T_{\theta }\left(V_{M}\right)\right)$$ (1)

The object function *ϕ* in Eq (1) measures the similarity between the fixed VOI *V*_*F*_ and the moving VOI *V*_*M*_. Multi-resolution registration is performed to enhance the registration robustness against ultrasound image texture variation [36]. Three intensity-based similarity metrics were examined to compare their effectiveness in aligning the 3DEVUS data.

Mean Square Error (MSE) computes the averaged pixel-wise intensity difference:

$$\begin{array}{c} MSE\left(V_{F},V_{R}\right)=\frac{1}{M\times N\times T}\sum_{i=1,j=1,k=1}^{M,N,T}{\left(V_{F(i,j,k)}-V_{R(i,j,k)}\right)}^{2} \end{array}$$ (2)

where *V*_*F*(*i*, *j*, *k*)_ and *V*_*R*(*i*, *j*, *k*)_ represent voxel intensities at position (*i*, *j*, *k*) in fixed volume *V*_*F*_ and registered volume *V*_*R*_ respectively. *M* × *N* × *T* is the total number of voxels in the VOI. *MSE* is based on the assumption that the pixel intensity of the same homologous point remains constant. Smaller *MSE* indicates more precise registration.

Normalized Cross Correlation (*NCC*) computes pixel-wise cross correlation normalized by the square root of the autocorrelation of the two volumetric images:

$$\begin{array}{c} NCC\left(V_{F},V_{R}\right)=\frac{\sum_{i=1,j=1,k=1}^{M,N,T}V_{F(i,j,k)}\times V_{R(i,j,k)}}{\sqrt{\sum_{i=1,j=1,k=1}^{M,N,T}V_{F(i,j,k)}\times \sum_{i=1,j=1,k=1}^{M,N,T}V_{R(i,j,k)}}} \end{array}$$ (3)

*NCC* is between 0 and 1. *NCC* equal to 1 means that the two volumes have a linear relationship between intensities and thus indicates an optimal alignment. Misalignment leads to a smaller *NCC* value.

Mutual Information (*MI*) measures how much information that a random variable captures about another random variable. *MI* is calculated based on the entropy of each random variable. Mutual information between two random intensity variables of two volumetric images *I*_*F*_ and *I*_*R*_ is defined as:

$$\begin{array}{c} MI\left(I_{F},I_{R}\right)=H\left(I_{F}\right)+H\left(I_{R}\right)-H\left(I_{F},I_{R}\right) \end{array}$$ (4)

where *H*(*I*_*F*_) and *H*(*I*_*R*_) are the entropies of random variables *I*_*F*_ and *I*_*R*_ respectively. *H*(*I*_*F*_, *I*_*R*_) is the joint entropy of *I*_*F*_ and *I*_*R*_. From our experiments, *NCC* gave the best registration results and was used on all datasets. A quantitative comparison of these three metrics is presented in Results.

Fig 4 demonstrates the stage 1 registration results of one dataset. To assist visual comparison, the pubic bone and muscle boundaries were manually outlined at the corresponding imaging plane. Fig 4B and 4C show one image slice in the fixed dataset D3 and one in the moving dataset D1, respectively, before registration. Misalignment of the two pre-registration volumetric images was obvious because the corresponding bone or muscle boundaries were apart from each other in Fig 4E. Note that the manually segmented pubic bone and muscle boundaries were only for the ease of visual comparison. The algorithm itself does not use edge features at all. Fig 4D shows the resampled image slice in D1 after registration at the same imaging plane as Fig 4B. Fig 4F shows that after registration, alignment of the pubic bone was significantly improved. This was demonstrated by the proximity of the pubic bone boundaries. However, the pelvic floor muscle regions were still misaligned (Fig 4F). The dissimilarity can be explained by the biased registration problem mentioned above. As a result, a rotational error occurred in regions distant from the bone VOI, such as in the muscle band.

Even with remaining misalignment, the registration based on the pubic bone region did manage to provide an initial alignment of two datasets to account for the variable probe placements. The initial alignment is necessary for subsequent multi-regional registration.

### Stage 2: Multi-resolution and multi-regional volumetric registration

**Fig 5:**
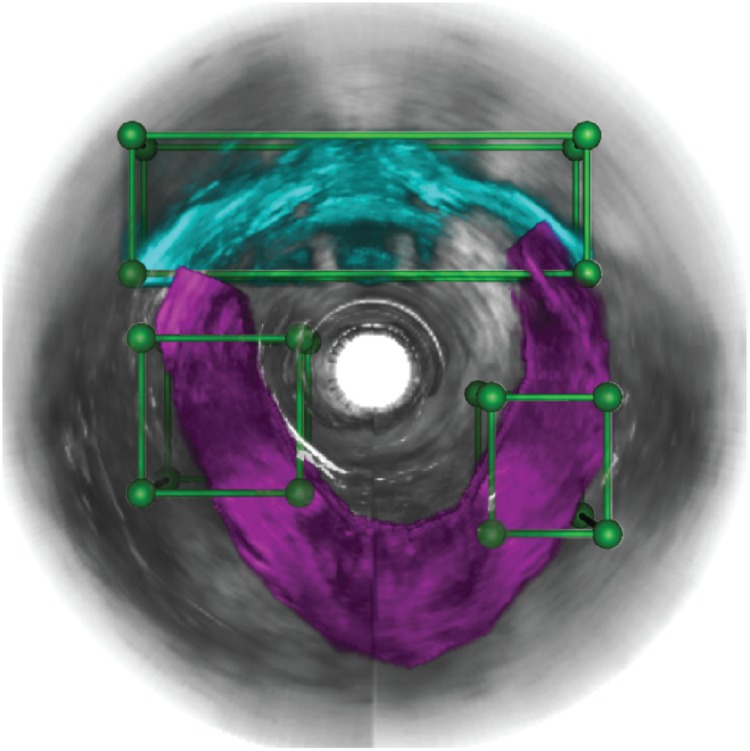
VOIs were defined near the pubic bone (cyan) and pelvic floor muscle (purple) regions. The green bounding boxes specified the volume of interests around the pubic bone (cyan) and pelvic floor muscle (purple).

We developed a weighted multi-regional registration scheme to refine alignment of the whole volume in the second stage to compensate for the registration bias due to the single and local VOI considered. Multiple VOIs that include the pubic bone ($V_{F_{PB}}$) and the pelvic floor muscle ($V_{F_{PM1}}$ and $V_{F_{PM2}}$) in the transferred moving volume D1 (Fig 5) were selected as the target VOIs. Because these regions are distributed in the volumetric data, they restrain each other from causing overfitting. Transformed D2 is defined as the moving volume and is transformed via registration to optimally align all target VOIs to those in the transferred D1. The weighted multi-regional registration is calculated by optimizing the cost function in Eq (5), which minimizes the weighted sum similarity *ϕ* between multiple VOIs ($V_{F_{PB}}$,$V_{F_{PM1}}$ and $V_{F_{PM2}}$) in the fixed volume *V*_*F*_ and the registered moving volume *T*_*θ*_(*V*_*M*_):

$$\hat{\theta }\;=\;\underset{\theta }{\underset{\theta }{\text{argmin}}\;\left(w_{1}\phi \left(V_{F_{PB}},T_{\theta }\left(V_{M}\right)\right)+w_{2}\phi \left(V_{F_{PM1}},T_{\theta }\left(V_{M}\right)\right)+w_{3}\phi \left(V_{F_{PM2}},T_{\theta }\left(V_{M}\right)\right)\right)}$$ (5)

*w*_1_, *w*_2_ and *w*_3_ are the weights applied on different VOIs which sum to one. These weights are introduced for two reasons: (1) ultrasound image pixel intensities of different anatomical structures such as bone and muscle are different, which influences the overall similarity measurement between two volumes. (2) the calculated similarity is also sensitive to VOI size, which is variable among selected VOIs. In order to manage impact of these factors, weight scalars are multiplied to the corresponding similarity measurements so that their contributions to the overall similarity can be adjusted. Values of the weights and sizes of the VOIs can be determined systematically through trial and error. The values leading to the best registration result are used.

When solving Eq (5), we adopted a multi-resolution implementation in order to overcome the lower ultrasound signal-to-noise ratio, heterogeneous ultrasound echotexture, and local optimum problems [54]. Multi-resolution approach is commonly used in image processing to improve an algorithm’s robustness against noise. The fixed image data is first resampled at different resolutions and the moving image is resampled at the coarsest level. Starting from the coarsest level, the transformation is optimized for the pair of datasets at this level and applied on the moving image. The resulting transformed moving image is then resampled at the second coarsest level that is used in optimizing registration at this finer level. The procedure is repeated until reaching the original resolution.

In terms of optimization, the adaptive stochastic gradient descent optimizer was used which converged faster for our data compared to other algorithms such as the Levenberg Marquardt optimizer. The optimization was performed for 2000 iterations on each level. Different numbers of multi-resolution levels were tested and three levels was chosen. Increasing the number of levels over three did not significantly improve the performance.

### Registration validation

We did cross validation to assess accuracy of M3VR. M3VR is intensity-based rather than feature based. We used identifiable features such as the anatomical structure boundary to cross-validate the results. Because these features were not used explicitly in the algorithm, which utilizes pixel intensity only, we hope such an assessment can be more objective and more powerful to expose any weakness of the proposed method.

**Fig 6:**
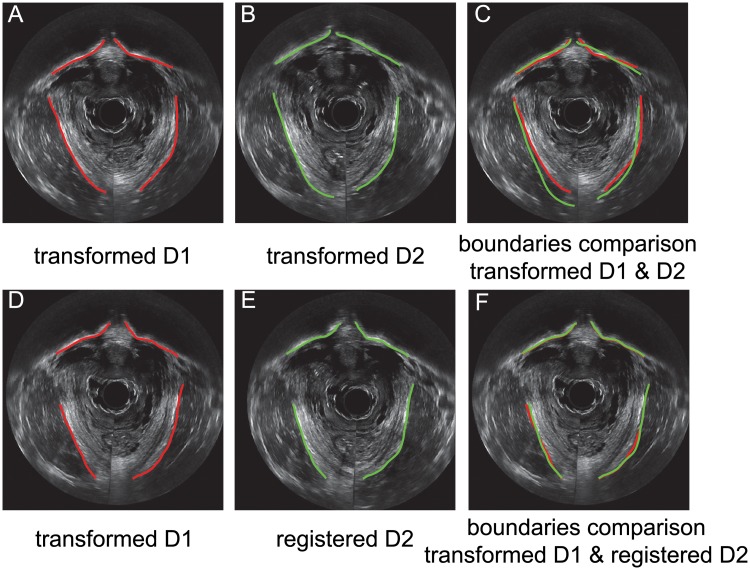
Pubic bone and muscle volumetric registration and validation after each stage. Before stage 2 registration, pubic bone and levator ani muscle boundaries are manually outlined in transformed D1 (A) and transformed D2 (B). The pubic bone boundaries show satisfying spatial proximity but the muscles misaligned (C). After stage 2 (D) and (E), bone and muscles boundaries were effectively aligned (F).

Pubic bone and pelvic floor muscle boundaries before and after applying M3VR were manually segmented and compared both visually and quantitatively. Fig 6 demonstrates how the validation was perform, with more results summarized in Results. Fig 6A and 6B show image slices at the same spatial location from transformed D1 and D2 after the first stage registration. Overlaid bone boundaries (Fig 6C) show satisfying spatial proximity but the muscles do not. This indicates overfitting of the bone region. Fig 6D and 6E show image slices from D1 and D2 at the same spatial location after applying the second stage registration, which was effective in aligning both the pubic bone and muscle regions (Fig 6F). The mean distance between the two sets of curves error is 0.65 mm.

## Results

We applied M3VR on Endovaginal ultrasound data of nine subjects. Registration accuracy evaluation was performed on each subject.

To test sensitivity of registration accuracy to VOI size, we experimented with the following volume sizes for the muscle VOIs: 10 × 10 × 10 *mm*^3^, 20 × 20 × 20 *mm*^3^, 20 × 30 × 20 *mm*^3^, 20 × 30 × 30 *mm*^3^, 20 × 30 × 40 *mm*^3^, 20 × 30 × 50 *mm*^3^, 20 × 30 × 60 *mm*^3^, and 20 × 30 × 70 *mm*^3^. The best values of the muscle VOI was found to be 20 × 30 × 30 *mm*^3^. The pubic bone VOI was finalized to be 25 × 37 × 12 *mm*^3^ being a bounding box of the pubic bone. The optimal weight values were determined to be *w*_1_ = 0.3, *w*_2_ = *w*_3_ = 0.35. These were obtained through systematically altering the weights between 0 and 1 with the constraint *w*_1_ + *w*_2_ + *w*_3_ = 1.

We used checkerboard validation, levator ani muscle boundary proximity measurement, as well as volume intensity similarity to quantitatively evaluate accuracy of the proposed method.

### Qualitative validation

**Fig 7:**
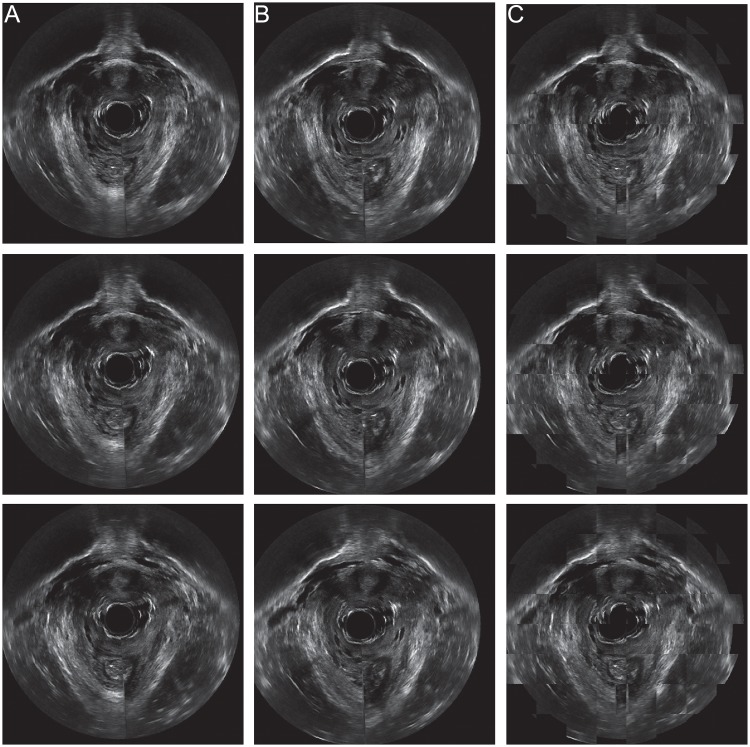
Checkerboard validation. (A) A sequence of fixed images in coronal view. (B) Moving images at the same imaging planes after 3D registration. (C) Checkerboard images of the two images on the corresponding rows, which show structure continuity and demonstrate effectiveness of M3VR.

Fig 7 illustrates registration results on one patient using the superimposed checkerboard images, which were used to for visual comparison of two registered images [55–58]. Fig 7A shows a sequence of image slices in the coronal view of the fixed image data. This includes the pubic bone and pelvic floor muscles at consecutive anatomical locations. Fig 7B shows the post-registration moving image at the same corresponding imaging planes. Comparing Fig 7A to 7B, we conclude that registration was able to transform the moving image to closely align with the fixed image. Fig 7C shows the checkerboard images generated from the corresponding fixed image slice and moving image slice in the same row. The smoothness of the checkerboard images further demonstrates the effective registration that results in good correspondence of anatomical structures in these two volumetric data. Boundaries of bone and muscle were continuous with minimal intensity difference. For instance, the pubic bone in the synthetized images in Fig 7C appears to be spatially continuous. Checkerboard validation was performed on all datasets. These all show satisfying visual alignment.

### Cross validation of M3VR accuracy

**Fig 8:**
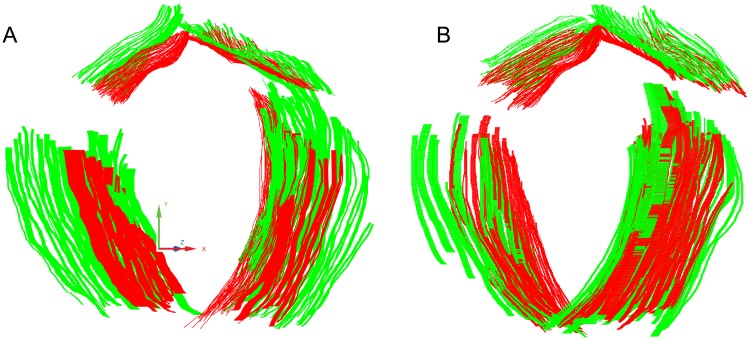
Comparison of pubic bone and levator ani muscles boundaries (A) before and (B) after applying M3VR. Green: boundaries from fixed ultrasound volume; red: boundaries from moving ultrasound volume.

We manually outlined boundaries of the pubic bone and levator ani muscle from the fixed and registered images (*B*_*F*_ and *B*_*R*_) to quantitatively evaluate the registration accuracy. Close proximity of the two sets of boundaries indicates effective registration. We use the Mean Distance Error (MDE) to evaluate the boundary proximity. After manual segmentation of the boundaries, 3D boundary points were calculated based on the spatial resolution of the volumetric data (Fig 8). For each boundary point belonging to the fixed image $p_{F}^{i}\in B_{F}$, *i* = 1, 2, …, *m*, we computed its shortest distance to the registered boundary *B*_*R*_. The proximity between the two sets of boundaries was then calculated as the mean shortest distance error averaged over all the m boundary points from the fixed image. A zero MDE means the two sets of boundaries overlap exactly and a large MDE indicates misalignment either translationally, rotationally, or both.

We validated our method on the 3D endovaginal ultrasound data from 6 patients that showed traceable levator ani muscle boundaries. The other 3 patients were subject to levator ani muscle deficiency resulting in loss of muscle volume, which degenerated its outer boundary visibility. Echotexture analysis can still be performed on the remaining muscle in these dataset. However, they were not suitable for registration accuracy assesssment because the mannual boundary segmentation might be subject to significant uncertainty resulting in an unfair comparison. Every 3D endovaginal data was converted into a sequence of 2D coronal images. Those images that contained clear appearance of the pubic bone and levator ani muscle were identifed. Structural boundaries were outlined in ImageJ [59] and then remapped to 3D. Fig 8 shows a visual comparison of one dataset. It can be seen that misalignment of both pubic bone and muscle boundaries (Fig 8A) were significantly corrected after registration. Whereas the muscle boundaries were nearly perfectly aligned, a small amount of misalignment of the bone boundary was observed in Fig 8B. Such error resulted from the variable local deformation of the pelvic floor soft tissue in individual datasets caused by replacement of the probe in the vagina, which was impossible to put the prob back to exactly the same location relative to the pubic bone. We had to make a tradeoff between optimally aligning either the bone VOI or the muscles VOIs by adjusting the weights in the objective function. We chose to associate higher values with the muscle VOI weights because accurate alignment of the muscle region is more critical for subsequent study on examining levator ani muscle echo texture in correlation with biopsy analysis.

We compare the registration results of the M3VR method with two other methods: symmetric block-matching [60] and global rigid registration [61]. Symmetric block-matching method uses block-matching to establish the spatial correspondences. The joint forward and backward transformation parameters are computed simultaneously. Tables 1 and 2 summarizes the quantitative comparison and cross validation results on data from 6 patients. The Mean Distance Errors of the pubic bone and levator ani muscle are presented. The average distances of the pubic bone and levator ani muscles all decreased dramatically after applying 3D registration. For the given dataset, M3VR method has the smallest registration error in both the levator ani muscle and pubic bone regions. The results demonstrated the necessity of registration to accurately locate the biopsied tissue, as well as the effectiveness of our approach. The maximum boundary distance error is less than 1 mm, showing the tight upper bound of the registration accuracy.

**Table 1:** Cross validation results on the registration accuracy of the levator ani muscle. MDE—Mean Distance Error (mm).

Subject	Before	After		
		Block Matching [60]	Rigid [61]	M3VR
**1**	14.07±11.27	2.67±2.25	1.56±1.01	**0.78±0.36**
**2**	23.75±10.81	2.07±1.90	3.39±2.34	**0.61±0.39**
**3**	53.91±8.10	2.24±1.36	4.28±2.84	**0.71±0.42**
**4**	3.87±2.31	2.34±0.78	0.87±0.57	**0.69±0.35**
**5**	14.53±9.49	2.53±0.94	2.74±1.89	**0.76±0.38**
**6**	28.31±10.13	1.36±0.97	3.66±3.04	**0.83±0.37**

**Table 2:** Cross validation results on the registration accuracy of the pubic bone. MDE—Mean Distance Error (mm).

Subject	Before	After		
		Block Matching [60]	Rigid [61]	M3VR
**1**	39.06±2.58	0.77±0.40	0.66±0.56	0.30±0.23
**2**	16.04±4.10	0.57±0.71	1.28±1.51	0.44±0.29
**3**	34.16±4.02	9.02±2.10	9.42±1.40	0.43±0.29
**4**	2.90±2.06	2.34±0.78	2.28±1.82	0.99±0.33
**5**	10.90±2.90	2.26±0.42	1.05±1.64	0.76±0.39
**6**	24.43±3.94	0.89±0.56	1.44±1.16	0.50±0.34

**Fig 9:**
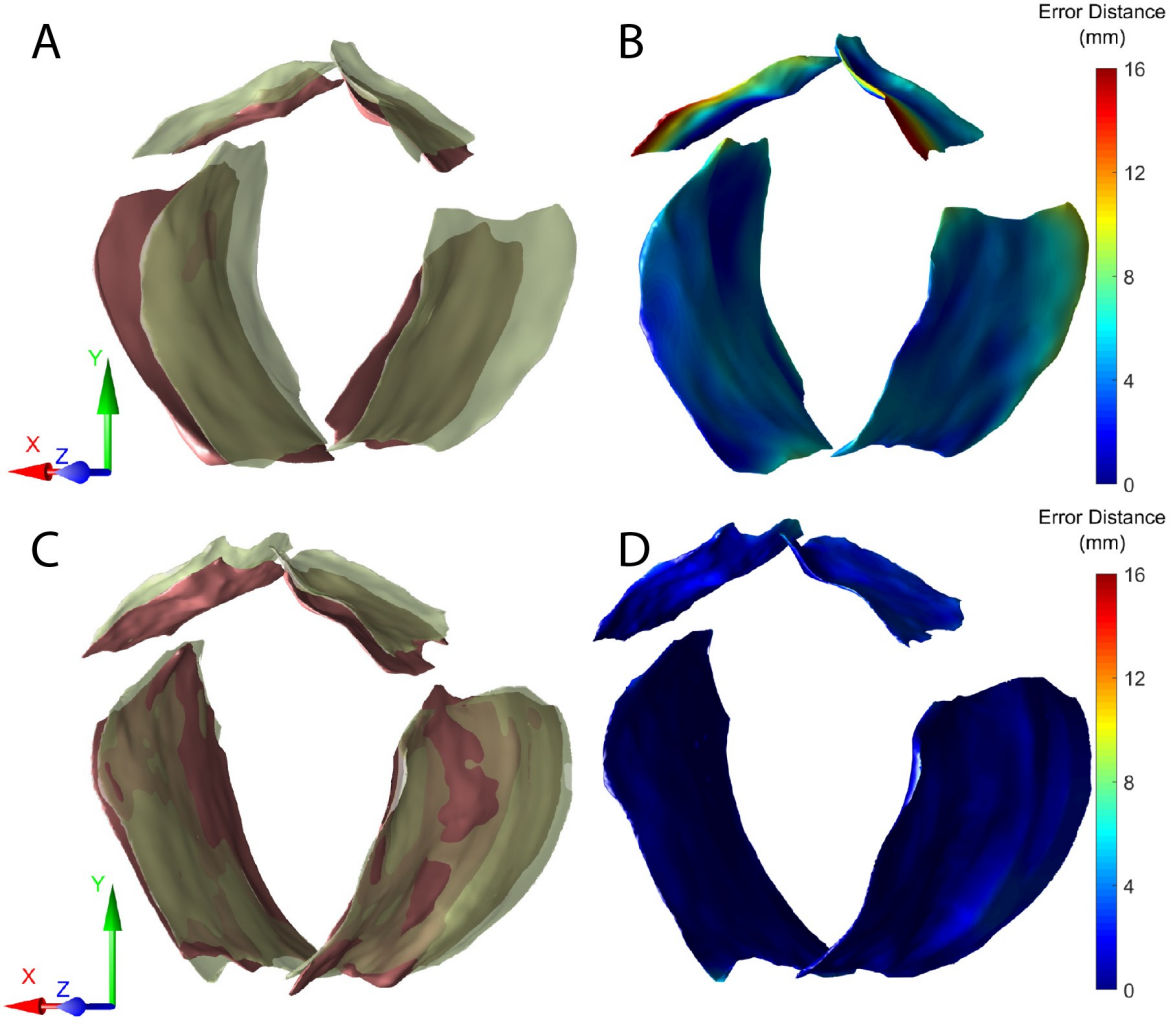
Comparison of the reconstructed pubic bone and levator ani muscle surfaces before and after applying M3VR. (A) Pubic bone and levator ani muscle surfaces before registration. (C) Pubic bone and levator ani muscle surfaces after registration. Red: surfaces reconstructed from fixed ultrasound volume; green: surfaces reconstructed from moving ultrasound volume. Visualization of one-side error distances from the reconstructed moving surface (green) to reconstructed fixed surface (red) before (B) and after (D) applying M3VR. Error distance are visualized using a blue-green-red colormap with blue corresponding to smaller error and red to larger error.

**Fig 10:**
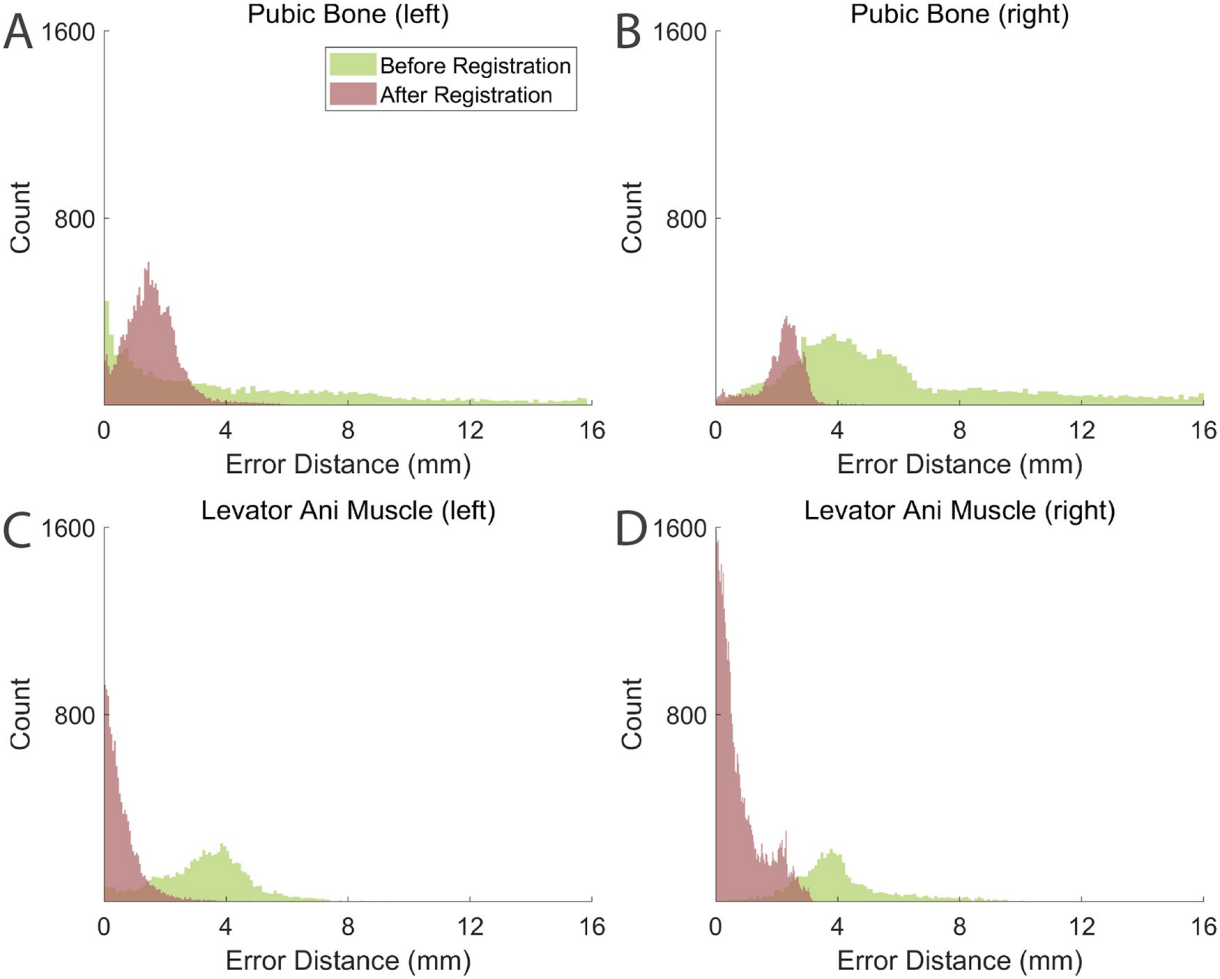
Comparison of histograms of error distance of pubic bone and levator ani muscle surface distances before (green) and after (red) applying M3VR. Histograms of error distance of (A) left and (B) right public bone. Histogram of error distance of (C) left and (D) right levator ani muscle surface distances.

We used the shortest distance map [62] which quantitatively measures the surface proximity to evaluate the spatial distribution of misalignment. Surfaces of the pubic bone and the levator ani muscle were first reconstructed from the 3D positions of the boundary points (Fig 9). We defined the one-sided error distance between a point *p*_*M*_ on the moving surface *S*_*M*_ (Fig 9—green) to the fixed surface *S*_*F*_ (Fig 9—red) as *d*(*p*_*M*_, *S*_*F*_):

$$\begin{array}{c} d\left(p_{M},S_{F}\right)=\min_{p_{F}\in S_{F}}E\left(p_{M},p_{F}\right),\;p_{M}\in S_{M} \end{array}$$ (6)

where *p*_*F*_ is the point on *S*_*F*_ that is closest to *p*_*M*_. *E*(*p*_*M*_, *p*_*F*_) is the Euclidean distance between points *p*_*M*_ and *p*_*F*_. The shortest distance map of a surface encodes the one-sided errors of all points on it. A shortest distance map associated with small values indicates the closeness of two surfaces whereas a shortest distance map with large values reflects greater misalignment. Fig 9 compares the shortest distance maps of one dataset before and after registration. Both pubic bone and levator ani muscle were misaligned before registration (Fig 9A), shown by the blue-green-red colormap (Fig 9B). The pubic bone had larger error compared to the levator ani muscle before registration. After applying M3VR, the two surfaces were much closer to each other (Fig 9C) as shown by the bluer shortest distance map (Fig 9D). We also compared the histograms of the one-sided error distance before and after registration to further analyze the corrected misalignment (Fig 10). The error distances of the pubic bone and levator ani muscle significantly decreased after applying M3VR.

Table 3 shows the mean error of each anatomical structure. The levator ani muscles have smaller mean error distance compared to the pubic bones. This indicates that M3VR was able to effectively improve the alignment in the levator ani muscle regions as well as reduce the overall misalignment at different spatial regions.

**Table 3:** Comparison of mean SDM on the registration accuracy.

Region		Before	After
**Pubic bone**	Left	4.47±4.14	**1.58 ±0.88**
	Right	5.96±3.69	**2.16 ±0.74**
**Levator ani muscle**	Left	3.28±1.49	**0.61 ±0.62**
	Right	4.07±1.61	**0.78 ±0.75**

### Assessment of similarity metrics

Different volume intensity similarity metrics including mean squares error, normalized Cross Correlation, and mutual information have been applied in different medical image analysis tasks. As mentioned earlier, we chose normalized cross correlation to measure similarity of the fixed image and the moving image. The decision was made after systematically examining the influence of different metrics on the registration results. Table 4 summarizes our analyses. These results will be potentially informative for future studies on volumetric ultrasound registration. Similarity between the fixed image and the moving image measured by each of the metric before and after registration was calculated from all datasets. Consistent with the cross validation results on boundary proximity, registration using any of these similarity metrics in the objective function was able to effectively improve alignment of two volumetric data sets.

**Table 4:** Quantitative analysis of the registration results using volume intensity similarity. Mean Square Error (MSE), Normalized Cross Correlation (NCC), and Mututal Information (MI).

	MSE		NCC		MI	
Subject	Before	After	Before	After	Before	After
**1**	2.52	**0.69**	0.85	**0.98**	1.38	**2.28**
**2**	14.35	**4.99**	0.80	**0.96**	0.96	**2.31**
**3**	26.96	**8.62**	0.81	**0.96**	1.26	**2.21**
**4**	12.41	**1.85**	0.71	**0.95**	0.83	**2.21**
**5**	28.93	**2.22**	0.63	**0.95**	0.72	**2.32**
**6**	37.69	**16.35**	0.70	**0.94**	0.34	**2.24**
**7**	18.28	**6.06**	0.83	**0.95**	1.31	**2.22**
**8**	36.72	**13.53**	0.81	**0.96**	0.63	**2.30**
**9**	20.93	**5.95**	0.77	**0.95**	0.56	**2.30**

**Fig 11:**
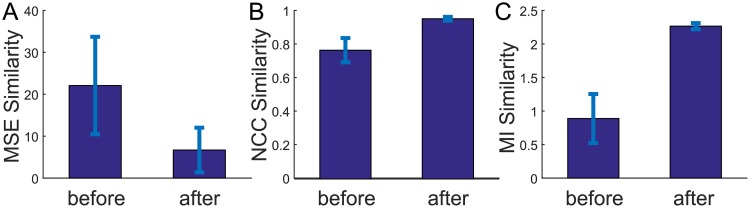
Comparison of intensity similarity measurement of the three metrics before and after registration. The mean and standard deviation of registration accuracy are calculated through mean square error (A), normalized cross correlation (B) and mutual information score (C). The registration accuracy is significantly improved after applying M3VR registration.

Fig 11 compares mean and standard deviation of the registration accuracy in Table 4. Mean square error score deceased from 22.10 ± 11.62 to 6.69 ± 5.33 (*p* <.001) after registration. Normalized cross correlation score increased from 0.77 ± 0.07 to 0.96 ± 0.01 (*p* <.0001) and mutual information score increases from 0.89 ± 0.37 to 2.27 ± 0.05 (*p* < 0.0001). All three scores show statistically significant alignment improvement after registration.

### Computational time analysis

**Fig 12:**
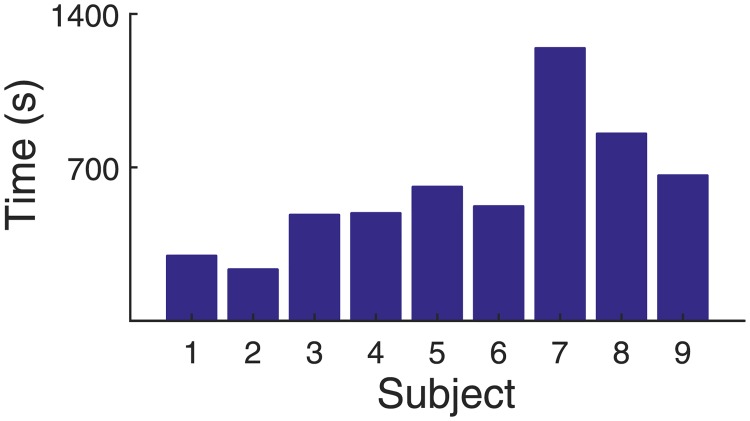
Computational time of M3VR for different subjects. The computational time of M3VR varies among different subjects. The time difference is mainly affected by sizes of the VOIs and the quality of initial registration.

The computational time of M3VR is mainly affected by sizes of the VOIs and quality of the initial registration. The closer the two volumes are after the initial registration, the less time it takes to converge to the minimum in the second stage. The overall registration time of M3VR on the night datasets included in our study took between 234.20 to 1243.73 seconds of CPU time on a Dell T7600 workstation (Fig 12). Considering the size and resolution of these datasets, we conclude that M3VR is computational affordable for offline volumetric registration.

## Discussion and conclusion

In this study, we developed and validated M3VR, a volumetric image registration approach to optimally align multiple ultrasound data sets containing anatomical differences. The main contribution is that the method is able to optimally align volumetric datasets that are subject to significant variation at multiple locations. Our results on 3D endovaginal ultrasound data from women undergoing surgical biopsy demonstrated the feasibility and effectiveness of the proposed method. With minor modification on the similarity measurement, M3VR can be applied on registering 3D or 2D data of other medical imaging modalities, such as MRI and CT. 3D to 3D, 2D to 2D, 2D to 3D or 3D to 2D registrations can all be formulated in our framework. Furthermore, M3VR can perform inter-imaging modality registration and inter-subject registration.

M3VR does not require any image intensity threshold to be specified but relies on the raw intensity values to find the optimal alignment. The only user inputs are size and location of each VOI. From the literature and our experiment, these parameters play an important role in the registration performance. Therefore, similar to many other registration approaches, parameter tuning is a necessary step to ensure the best results. What we found was that the optimal parameter values were fairly consistent across different datasets. Therefore, there is no need to fine-tune VOI parameters for every dataset but only for one or a few representatives. Furthermore, the process can be automated by systematically varying the parameter values within a reasonable range in the computer program while exporting the registration accuracy of each case for offline analysis. The program can be set to test all feasible parameter sets in one run. Then the optimal parameter values associated with the smallest error can be identified and used for all other datasets.

We experimented with both feature-based and intensity-based registration. We decided to perform intensity-based registration. The main reason of using image intensities and not features was that it was difficult to robustly and automatically extract anatomical features of the inner boundary of the pubic bone and outer boundary of the levator ani muscle from all datasets. The advantage of intensity-based registration is that potential error or parameters involved in feature extraction are avoided. The limitation is that many intensity-based similarity metrics such as mean square error and the adopted normalized cross correlation, implicitly assume pixel-to-pixel intensity value correspondence. This may not hold even in the optimal alignment of medical image data [63]. Although our study showed that non-pixel-wise similarity metrics such as mutual information did not lead to better results, future studies may consider other similarity metrics suitable to compare texture characteristics in the proposed framework. For instance, histograms of the pixel intensity values emphasize the high level structural property of the VOI rather than low level individual pixel intensity values and can be used in the objective function to characterize underlying structures [64].

As mentioned earlier, the pelvic floor volume being scanned was completely rigid mainly due to the removal and reposition of the ultrasound probe. However, our method did assume rigid relationships among the multiple VOIs, which unavoidably introduced error in similarity measurement. Future work may consider a hybrid approach that allows affine transformation between any two VOIs while assuming rigidity of each VOI. Spatial relationships of the VOIs can be used as priors to initialize and constrain their relative transformation to avoid overfitting if all VOIs are registered independently.

Implemented in C++, M3VR took up to 21 minutes to register two high resolution volumetric datasets (800 × 800 × 650). Although it is not as fast as some of the existing volumetric registration algorithms that are able to complete in seconds or a few minutes [25, 32, 35], it is still practical for many offline analysis applications. Future work on improving the computational efficiency of M3VR will be focused on reducing irrelevant volume for processing and utilizing the paralleling computing advantage of GPU [11, 25] and more powerful computer workstations. This will significantly reduce the computational time to determine model parameters and produce optimal alignment for each dataset while maintaining the same accuracy, even if the computations are not in real time. With further improvement, M3VR can potentially become a useful advanced registration algorithm for many biomedical applications.
